# Translation and validation of a Swedish version of the Visual Vertigo Analogue Scale

**DOI:** 10.1080/07853890.2023.2177724

**Published:** 2023-03-09

**Authors:** Tobias Wibble, Tony Pansell

**Affiliations:** a Department of Clinical Neuroscience, Division of Ophthalmology and Vision, Marianne Bernadotte Centre, Karolinska Institutet, Stockholm, Sweden; b St. Erik Eye Hospital, Stockholm, Sweden

**Keywords:** Visually Induced Dizziness, vertigo, visual vertigo analogue scale, Swedish visual vertigo analogue scale, VVAS, VVAS-S

## Abstract

**Purpose:**

The present study aimed to construct and validate a Swedish translation (VVAS-S) of the Visual Vertigo Analogue Scale (VVAS).

**Materials and Methods:**

The original English VVAS was translated into Swedish by the two authors and back-translated by an independent professional translator. Pilot-tests were performed on two healthy participants and five patients suffering from Visually Induced Dizziness (VID). The translation was deemed understandable by all subjects. Twenty-one patients with VID were recruited to complete the VVAS-S, once in-lab and once at home after 2–3 weeks. Cronbach’s alpha, inter-item consistency and internal consistency were calculated.

**Results:**

Test-retest values were reliably strong across all items. Cronbach’s alpha was 0.843, which is considered to represent very-high reliability. The corrected-item total-correlation was above 0.3 for all items, meaning they were appropriately associated with one-another. Fourteen out of 36 inter-item correlation interactions were within the 0.2–0.4 range.

**Conclusions:**

The VVAS-S was found to be comparable to the original VVAS in terms of internal reliability. The translation was perceived as easy to implement by all participants and can be considered ready for clinical use in a Swedish-speaking setting. Item-specific correlations may be valuable for developing future vertigo questionnaires.

**Key messages**
The Swedish version of the Visual Vertigo Analogue Scale is a questionnaire suitable for evaluating visually induced dizziness in a Swedish population. This study found that the Swedish questionnaire was comparable to the original in terms of internal consistency. The Swedish Visual vertigo Analogue Scale can be found as an appendix to this article.

## Introduction

Vertigo is one of the most common complaints in the emergency room [1]. Despite its prevalence the condition remains difficult to classify and there is a lack of evaluation methods available to clinicians [1,2]. As it stands, the primary means of diagnosis is through testing the peripheral vestibular system [3]. However, cirka half of all vertigo patients express no clear pathology and are often discharged with no clear follow-up [1]. As vertigo is a subjective sensation, subjective assessment scales offer valuable tools for grading vertigo symptoms. The Visual Vertigo Analogue Scale (VVAS) was developed by Dannenbaum et al. as a derivative of the Longridge et al. aiming to identify patients with a visual aethiology for their complaints [4].

The widely accepted hypothesis for why vertigo and motion sickness arise has its basis in the sensory mismatch theory [5]. A stable sensation of balance relies on matched signals from visual, vestibular, and somatosensory systems, which are all integrated seamlessly at the central level. Visual vertigo, or Visually Induced Dizziness (VID), is a set of symptoms where patients are believed to have developed visual dependency following a vestibular pathology, and as a result respond with discomfort and hypersensitivity to visual clutter or motion [6]. VID can also be caused by a range of other conditions, most notably concussion and migraines, but the condition can also be seen in Parkinson’s disorder, stroke, and neuropsychiatric disorders [7–11].

The English VVAS has been used for clinical [12] and academic [13] evaluations relating to symptoms of VID. It has been successfully adapted into Spanish [14] and Arabic with high internal consistencies [15]. Operating in Stockholm, Sweden, we have utilized the English VVAS in our clinical work. However, we have found that several items require additional explanation as it is perceived as containing specialized language. Introducing a Swedish VVAS (VVAS-S) would offer a clinical tool that can be easily implemented throughout the Swedish healthcare system.

This study aimed to translate and validate a Swedish version of the Visual Vertigo Analogue Scale. This was done through forward and backward translations of the English questionnaire, followed by an evaluation performed by an expert committee, allowing pilot testing with subsequent revision. The resulting questionnaire was then validated in terms of internal consistency and intraclass correlations to assure it meets the standards established by the original English version.

## Methods

### Translation process

The translation and validation process followed a well-established methodology; this involved making preliminary considerations for the creation of the Swedish VVAS (VVAS-s; see Supplementary appendix 1), producing a formal translation of the questionnaire, and going through the validation process [16]. After having identified the need for a Swedish VVAS, an expert committee was established. This committee consisted of the two authors (TW and TP). Both are clinically active healthcare professionals, meet patients with vertigo symptoms, and are active researchers within the field.

After receiving written permission from the VVAS’s original author, Dr. Dannenbaum, to translate the original English version into a VVAS-S, each item on the questionnaire was forward translated from the original version into Swedish. This was done separately by both authors. The two versions were then compared, and any discrepancies discussed. This resulted in a preliminary version of the VVAS-S. This version was sent to a professional, native-English speaking translator living in Sweden and backwards-translated into English. The back-translated version was compared to the original version by the authors. No considerable deviations were identified, and the preliminary version was kept.

Pilot testing of the VVAS-S was done in two healthy subjects and five patients suffering from VID following long-lasting symptoms from concussion. All seven subjects had Swedish as their native language. After giving oral instructions about how to complete the questionnaire, participants answered each item in their own time, completing the questionnaire in approximately 5 minutes. Each subject was then interviewed about the meaning of each item to discover any misinterpretations of the included items, and was offered the opportunity to provide feedback. Altogether, pilot participants considered the VVAS-S to be easily understandable, and no changes were carried out.

### Validation process

Thirty patients suffering from concussion and visual vertigo were invited to participate in the study and were asked to complete the VVAS-S questionnaire. Inclusion criteria included current VID complaints as evaluated by at least one independent clinician with experience in the field, Swedish as a first language, and being aged 18-55 so as to preclude presbyvertigo. Exclusion criteria included having recently started medication influencing balance or the central nervous system, a vestibular or neurological diagnosis, or having suffered a concussion within 3 months or later than 12 months; this time range minimized the risk of additional symptoms or complications from acute or complex long-term brain trauma that may have confounded the symptomology. Ultimately, all participants were patients referred from local physiotherapy clinics due to VID following concussion. All participants completed the VVAS-S twice.

All participants received written and oral information on the procedure, after which they provided written consent prior to their enrolment. The research complied with the Declaration of Helsinki and was approved by the Regional Ethics Committee of Stockholm (EPN 2018-1768-31-1). Participants were offered to participate in the study after having completed their planned visit, and were informed that their participation would in no way interfere with their rehabilitation.

### Statistical analysis

Reliability analysis of the VVAS-S was evaluated by calculating the Cronbach’s alpha coefficient. Internal consistency for individual items on the VVAS-S evaluated how well each item measured visual vertigo and is regarded as satisfactory if *α* > 0.70, and good if *α* > 0.80. The coefficient was also calculated after each item had been deleted to establish how it affected the instrument’s reliability. Test-retest data from 21 patients were used to calculate retest reliability using the intraclass correlation coefficient (ICC) by calculating Spearman non-parametric correlational coefficients. This value measured the strength and direction of association between the within-subject responses given at the first and second occasion. Correlations were judged to be significant at *p* < .05, and the strength of the coefficients were regarded as follows: small (0.1–0.29), mild (0.3–0.5), moderate (0.5–0.7), and strong (greater than 0.7). All statistical operations were carried out using IBM SPSS Statistics 25 for Windows.

## Results

Data from 21 patients with symptoms of Visually Induced Dizziness (VID) were included in the analysis; out of 30 patients invited, five rejected the invitation and four did not complete the second VVAS-S. The data was not normally distributed according to Shapiro-Wilk test and exhibited negative distribution according to skewness and kurtosis, meaning the data distribution was weighted towards higher symptom levels on the scale. A ceiling effect was observed in three patients (14.3%) in one or more items. No flooring effect was observed. Each subject answered the VVAS-S a second time, approximately two-three weeks after the first questionnaire. Total scores were a median of 58.9 for the first questionnaire, and 58.4 for the second questionnaire, with no significant differences between the two timepoints. The retest correlations were strong (*r* > .72). For details, see Table 1.

**Table 1.** Descriptive stats (median (quartile 1 : quartile 3) from each item and questionnaire (*n* = 21).

	VVAS 1	VVAS 2	Wilcox. Sign-rank test (sig.)	Test-retest Correlation (Spearman)
Item 1	6.2 (4.8: 6.9)	6.2 (4.1: 6.8)	*z* = 1.356 (n.s.)	0.788**
Item 2	6.6 (4.0: 7.8)	7.4 (5.1: 8.3)	*z* = 0.713 (n.s.)	0.722**
Item 3	6.4 (3.5: 8.1)	5.6 (4.2: 8.4)	*z* = 0.299 (n.s.)	0.826**
Item 4	6.4 (4.3: 7.6)	5.8 (4.2: 7.4)	*z* = 0.187 (n.s.)	0.797**
Item 5	6.9 (5.5: 7.9)	6.8 (5.0: 7.8)	*z* = 0.896 (n.s.)	0.751**
Item 6	4.2 (2.8: 6.3)	4.9 (3.5: 6.5)	*z* = 0.608 (n.s.)	0.789**
Item 7	7.7 (6.0: 9.1)	7.9 (5.9: 8.7)	*z* = 0.299 (n.s.)	0.804**
Item 8	6.0 (4.6: 8.5)	5.5 (3.2: 7.8)	*z* = 0.434 (n.s.)	0.739**
Item 9	8.5 (7.3: 9.0)	8.3 (6.3: 9.1)	*z* = 0.747 (n.s.)	0.777**
Median score per item	6.4 (6.2: 6.9)	6.2 (5.6: 7.4)		

The Median total score was 54.4 (46.7: 66.0) for VVAS 1 and 50.9 (48.1: 70.6) for VVAS 2. No significant differences were noted while a high test-retest correlation was observed. ** = *p* < .01.

Additional evaluations of the questionnaire were performed in order to ensure that the Swedish VVAS offered comparable symptom assessments to the original version. An inter-item consistency matrix was constructed to assess the internal consistency of the VVAS-S. The data set from the second VVAS-S was used so as to allow each participant to fill in the questionnaire in a relaxed setting in their own home and with minimal external influence on their grading. For details about the inter-item internal consistency, see Table 2. The overarching consistency was evaluated using the Cronbach’s alpha, which for the VVAS-S was found to be 0.843. Item discrimination in terms of Corrected Item-Total Correlation (CI-TC) was calculated for each item to evaluate to what extent each item could be associated with other items in the questionnaire. Item 7 displayed the lowest CI-TC value, and Cronbach’s alpha was slightly improved if item 7 was deleted. For details, see Table 3.

**Table 2.** Inter-item internal consistency of the VVAS-S.

	Item 1	Item 2	Item 3	Item 4	Item 5	Item 6	Item 7	Item 8	Item 9
Item 1	1	0.543	0.511	0.242	0.742	0.417	0.451	0.436	0.458
Item 2		1	0.433	0.305	0.185	0.446	0.151	0.130	0.380
Item 3			1	0.575	0.604	0.632	0.121	0.651	0.412
Item 4				1	0.357	0.603	0.106	0.278	0.271
Item 5					1	0.428	0.373	0.426	0.478
Item 6						1	0.173	0.392	0.168
Item 7							1	0.169	0.507
Item 8								1	0.088
Item 9									1

Internal consistency between items were evaluated through an inter-item internal consistency matrix, where the ideal range is deemed to be between 0.2 and 0.4 to be neither unfitting nor superfluous.

**Table 3.** The internal consistency of the Swedish VVAS.

	CI-TC	α if item deleted
Item 1	0.715	0.813
Item 2	0.461	0.840
Item 3	0.771	0.799
Item 4	0.520	0.830
Item 5	0.657	0.816
Item 6	0.636	0.817
Item 7	**0.334**	**0.847**
Item 8	0.485	0.835
Item 9	0.492	0.834

CI-TC Corrected Item-Total Correlation; α: Cronbach’s alpha.

Corrected Item-Total Correlation (CI-TC) was calculated for each item. A value of <0.3 can be regarded as grounds for removal from a questionnaire, as it indicates that the item may not be associated with the other statements. In addition, the Cronbach’s alpha was recalculated for the removal of each item. The Cronbach’s alpha for the intact questionnaire was 0.843.

## Discussion

The aim of this study was to prepare and evaluate the internal consistency of a Swedish translation of the Visual Vertigo Analogue Scale for use in a Swedish-speaking environment. The VVAS-S was translated according to international guidelines, evaluated by experts and novice users, and was proven to retain a high internal consistency among Swedish patients suffering from Visually Induced Dizziness.

While VVAS has traditionally been ascribed a vestibular pathogenesis [6,17], the condition is present in a range of conditions [7,8,18–22]. The present study enrolled patients primarily suffering from VVAS as part of their post-concussion syndrome. Several studies have shown VVAS to be a consistent sequelae of brain trauma, ranging from mild to moderate concussions [23–28]. The patient group in the present study differed from the original article by Dannenbaum et al., where patients were experiencing symptoms primarily due to vestibular pathologies, and the VVAS-S could therefore offer a useful illustration of VID induced by concussion. The Cronbach’s alpha of 0.83 can be interpreted as representing very high reliability. While it is not as high as the original score retrieved by Dannenbaum, 0.94 [4], it is comparable to the Arabic version of the VVAS, which enrolled vestibular patients and reached a Cronbach’s alpha of 0.83 [15]. We would therefore suggest that the VVAS is suitable for use in VID brought on by concussion, and that the Swedish translation meets a comparable standard so as to be considered of clinical utility.

In Dannenbaum’s study, seventy percent of these patients were women. This is comparable to the 66% enrolled in the present study, which was achieved as a reflection of the patient population and not by selection. This coincidental distribution is likely a reflection of the already described sex differences seen in patients presenting with VVAS after concussion [29]. As a point of difference, the mean score of Dannenbaum’s initial evaluation was 30.3, with a standard deviation of ± 26.7. The present study yielded higher mean scores with less variation: 55.91 (±12.51) and 55.10 (±14.32) for the first and second answer sheets respectively. While this reflects a very good reliability for the internal consistency of the VVAS-S, it invites the question of what may have caused the discrepancy in total scores between the English and the Swedish versions. One interpretation may be that concussed participants score higher than vestibular patients; this would naturally have to be assessed through a follow-up study comparing the two patient populations. A second alternative would be that the Swedish VVAS inadvertedly prompted patients to rate their symptoms higher; this could have been due to different associations patients may have had with certain items of the questionnaire, brought on by either linguistic or cultural differences between English and Swedish users. Considering the relatively small population size, and the differences in the patient population between the original and present studies, it is difficult to draw any concrete conclusion at this point. One may also note that the intraclass correlation coefficient (ICC) showed excellent reliability for item #6 and strong reliability for all other statements in the questionnaire. Regarding cross-cultural differences, a validation study of the thematically related Dizziness Handicap Inventory between English and Swedish noted a lack of questions relating to different methods of public transportation in the Swedish version, likely due to higher usage of public transportation in Sweden compared to North America [30]. Similarly, the VVAS does not contain any item relating to public transportation, though no participants commented on this fact in the present study; the item concerning travelling as a passenger in a car may very well have been extrapolated to included buses, although this was not specifically investigated. Altogether, there were no specific questions relating to cultural ambiguity during the validation process.

In order to further evaluate the suitability of each item, as presented by the Swedish translation, an inter-item correlation matrix was constructed. The reason behind this comparison was the notion that a questionnaire with several inter-item correlations reflects symptom-appropriate questions relatable to the patient cohort and altogether strengthen the integrity of the form. This method of evaluating a questionnaire is well-described, and is used to test if a series of questions give appropriate and consistent results [16]. This study found one strong and eight moderate correlations. Fourteen out of thirty-six interactions were between 0.2 and 0.4. Correlations within this range may be viewed as appropriately homogenous, while values below this level suggests that those items may not be representative of the population’s symptomology, and higher values suggest unnecessary repetition [31].

The Swedish VVAS held items #6 and #2 as most applicable for patients' symptoms, with five and four in-range correlations respectively. Items #7 and #1 instead had only one interaction each. As no similar data is available for the original VVAS, it is difficult to discuss any differences that may have been brought on by the translation. This finding may however be useful for developing future questionnaires pertaining to vertigo or dizziness. It is also noteworthy that item #7 had the lowest CI-TC score in the VVAS-S. The value was nevertheless above 0.3, which may be considered the lower limit at which an item may correlate with others in a scale [32]. The Cronbach’s alpha was also only marginally improved by its removal. Many participants offered input on item #7, noting that they generally did not visit the cinema as it was deemed too symptom provoking, and they therefore found it difficult to provide a fair estimation. The item was still included in the VVAS-S as it is featured in the original version and participants’ feedback was not considered contingent on the translation of the text.

In conclusion, the Swedish translation of the Visual Vertigo Analogue Scale was shown to have a very high internal consistency and was perceived as easy to understand by patients. The VVAS-S can be accessed freely online and implemented in a clinical setting. While this study did not aim to evaluate the items of the original VVAS, the inter-item correlation matrix may offer some guidelines for developing future questionnaires on vertigo or dizziness.

## Supplementary Material

Supplemental Material

## Data Availability

Data is available upon reasonable request from the authors.
