# Supplementary material for: Translation and validation of a Swedish version of the Visual Vertigo Analogue Scale
Source: Ann Med. 2023 Mar 9;55(1):572–7. doi: 10.1080/07853890.2023.2177724 (PMC10795583; doi:10.1080/07853890.2023.2177724)
Supplement: Supplemental Material [file IANN_A_2177724_SM0660.pdf]

Karolinska Institutet

# Visuell Yrsel Analog Skala

Indikera nivån av yrsel du upplever under följande situationer. Markera med ett streck på skalan nedanför.
0 representerar ingen yrsel och 10 representerar mest yrsel

När du går genom gången i en livsmedelsaffär

0 |————————————————| 10

När du åker med som passagerare i en bil

0 |————————————————| 10

När du befinner dig under lysrör

0 |————————————————| 10

När du tittar på trafiken vid en livlig gatukorsning

0 |————————————————| 10

När du går genom ett köpcenter

0 |————————————————| 10

När du åker nedför en rulltrappa

0 |————————————————| 10

När du ser på film på en biograf

0 |————————————————| 10

När du går över ett mönstrat golv

0 |————————————————| 10

När du tittar på TV med mycket bildrörelse

0 |————————————————| 10

Originalreferens för Visual Vertigo Analogue Scale: Dannenbaum E, Chilingaryan G, Fung J. Visual vertigo analogue scale: an assessment questionaire for visual vertigo. J. Vestib Res. 2011;21(3):153-9.
Översättning till svenska av Tobias Wibble och Tony Pansell, Marianne Bernadotte Centrum, Avdelningen för Ögon och Syn, Karolinska Institutet, Stockholm.
